# Intestinal polyparasitism with special emphasis to soil-transmitted helminths among residents around Gilgel Gibe Dam, Southwest Ethiopia: a community based survey

**DOI:** 10.1186/s12889-016-3859-2

**Published:** 2016-11-23

**Authors:** Zeleke Mekonnen, Sultan Suleman, Abdissa Biruksew, Tamirat Tefera, Legese Chelkeba

**Affiliations:** 1 Department of Medical Laboratory Sciences and Pathology, College of Health sciences, Jimma University, Jimma, Ethiopia; 2 Department of Pharmacy, College of Health Sciences, Jimma University, Jimma, Ethiopia

**Keywords:** Polyparasitism, Soil-transmitted helminths, Prevalence, Gilgel Gibe, Jimma, Ethiopia

## Abstract

**Background:**

One third of the world population is estimated to be infected with intestinal parasites. The most affected people are children and the poor people living in tropics and subtropics. Polyparasitism (the concurrent infection with multiple intestinal parasite species) is found to be the norm among the same population although accurate estimate of its magnitude is unknown. It was found that polyparasitism might have a greater impact on morbidity than single species infection which might also increase susceptibility to other infections. Therefore, this study aimed at determining the prevalence and distribution of intestinal polyparasitism with special emphasis on Soil-Transmitted Helminths (STH) among residents around Gilgel Gibe dam located in Jimma zone of Oromia regional state, Ethiopia.

**Methods:**

A total of 1,021 participants were recruited in this study and provided stool samples for parasitological examination. Direct wet mount and Kato-Katz techniques were employed for stool examination. Pearson chi-square test was employed to assess the association of infection status and polyparasitism with gender and age group of the study participants.

**Results:**

Five hundred thirty two individuals were infected with at least one parasite, providing the overall prevalence of 52.1%. Among positive individuals, 405 (76.1%), 114 (21.4%), and 13 (2.5%) individuals were infected with only one, two and three species of parasites, respectively. The overall prevalence of intestinal polyparasitism observed among the study participants was 12.4% (127/1,021). The predominant STH was hookworm, with a prevalence of 44.1%. Hookworm and *Ascaris lumbricoides* were the most frequently recorded combination in cases of polyparasitic infection. The study revealed that there was no significant difference in the distribution of polyparasitism with regard to age group and sex of the study participants (*p* > 0.05).

**Conclusion:**

The study indicated the presence of high prevalence of parasites as well as distribution of polyparasitism in the area. Moreover, the detection of *Schistosoma mansoni* in the community living within close proximity of the newly constructed dam would be taken as an indication of future risk factor. Further investigation on the predictors of polyparasitism and the assessment of effects of polyparasitism on the population are needed. Finally, there is a need to undertake integrated control strategies which involve improved sanitation, health education and chemotherapy that targets the whole community instead of only certain segments of populations.

## Background

Intestinal parasitic infections (IPIs) remain one of the most public health problems where one third of the world population is generally estimated to be infected. The most affected people are children and the poor people living in tropics and subtropics, where there is poor personal hygiene and environmental sanitation, overcrowding, and limited access to clean water [1, 2]. A concurrent infection with more than one parasitic species is also commonly observed among the same population [3].

The major IPIs affecting humans are the protozoan species (*Entamoeba histolytica/dispar* and *Giardia lamblia)* and helminthes (*Ascaris lumbricoides*, *Trichuris trichiura*, hookworms, *Strongyloides stercolaris, Enterobius vermicularis*) and other parasites (*Hymenolepis nana*, and *Schistosoma mansoni*). Among these IPIs, soil-transmitted helminthiasis and schistosomiasis are the most neglected tropical parasitic diseases disproportionately affecting the poor and most vulnerable populations with under-privileged living conditions. More than 1.5 billion people, 24% of the world population, are infected with soil-transmitted helminths (STHs) [4] causing 4.98 million years lived with disability [5]. As a result, about 300 million people suffer from severe morbidity attributed to STH infections, resulting in 10,000–135,000 deaths annually [6]. It is also estimated that 207 million people are infected with schistosomiasis worldwide, leaving 779 million people at risk of infection; the highest prevalence occurring in sub-Saharan Africa [7].

Although accurate estimate of the magnitude of polyparasitism (the concurrent infection with multiple intestinal parasite species) is unknown, the case is distributed widely in the tropics and sub-tropics [3]. As opposed to other pathogens, morbidity due to STH and schistosomiasis is related to the intensity of infection rather than the absence or presence of the parasites. It was found that individuals with polyparasitic infection also harbor the most intense infections [8, 9]. Even low-intensity of polyparasitic infections may result in clinically significant morbidity [10, 11]. Therefore, polyparasitism may have a greater impact on morbidity than single species infection. It may also increase susceptibility to other infections and adversely affect the clinical outcome of the concomitant diseases such as tuberculosis, HIV/AIDS and malaria [3, 12].

Even though the cases of polyparasitism are prevalent, few studies have considered its significance. Nevertheless, there is now increased emphasis on using a multispecies control approach as a cost-effective mechanism to control various helminth infections [9, 13]. In Ethiopia, IPIs are recognized as a major public health problem [14, 15]. The burden and magnitude of IPIs in Ethiopia varies according to geographical location, climate and nature of soil, the segment of the population studied and other related socio-demographic and socio-economic factors [16–18].

The construction of hydroelectric dam in the area could be among the factors enhancing the transmission of schistosomiasis in a given area by facilitating the breeding sites for the intermediate host (snails). Given the already existing burden of STH, this might create the opportunity for polyparasitic infection with special emphasis on STHs and *S. mansoni*. Therefore, this study was designed to determine the prevalence and distribution of intestinal polyparasitism among residents around Gilgel Gibe Dam.

## Methods

### Study area and setting

The study was conducted in the Gilgel Gibe hydroelectric power dam catchment areas found at 10 km away from the periphery of the dam which are found in four districts (Omo Nada, Tiro Afata, Sekoru and Kersa) of Jimma zone in the Oromia national regional state to the south west part of Ethiopia. It included 13 Gotes/villages from a total of 7 rural Kebeles (the smallest administrative unit in the country) of the four districts. The study area comprised of 11,000 households with a total population of 50,000. The study area lies between latitudes 7^0^42′50″ N and 7^0^53′50″ N, longitudes 37^0^11′22″ E and 37^0^20′36″ E at an altitude of 1672–1864 m above sea level. Gilgel Gibe dam is located at about 260 km south-west of Addis Ababa, the capital of Ethiopia; and 55 km north east of Jimma town. The dam started operating in 2004.

### Study design

A cross-sectional community based parasitological survey was conducted among population living around the Gilgel Gibe dam catchment areas between November and December, 2009.

### Sample size and sampling techniques

Sample size was calculated using single population proportion formula; assuming expected prevalence of 50%, 95% confidence level, 5% margin of error, 10% non response rate, and design effect of 3. The sample size was finally calculated to be 1,190. A multi-stage cluster sampling technique was employed. Briefly, 13 villages were conveniently selected from the source population. The determined sample size was then proportionally allocated to the size of each of the 13 selected study villages. From each village households were randomly selected and all individuals within the selected households were included in the study.

### Parasitological methods

All stool samples were collected between 9 and 10 am each morning and then transported to Jimma University parasitology laboratory. Sample processing and microscopic examination took place within 4 h of collection by direct wet mount, and on the portion of the stool sample Kato-Katz thick-smear techniques was prepared simultaneously and examined microscopically. Briefly, the Kato-Katz thick smears were prepared following standard operating procedures (SOPs) as described by WHO (1991) on microscope slides using a square template with a whole diameter of 6 mm and depth of 1.5 mm, which is assumed to sample 41.7 mg of stool. All slides were examined by experienced laboratory technologists within 30–60 min of preparation for the presence of STHs and other parasites and then after overnight for *S. mansoni*.

### Data analysis

Data were coded, entered into, cleaned, and analyzed using SPSS for windows version 20.0. Frequencies were used for calculation of the prevalence of infection and polyparasitism. Pearson chi-square test was employed to identify any association of intestinal parasitic infection with gender and age group.

### Quality control

Re-reading of 10% of the slides randomly selected was performed by senior microscopist blinded to the results for assuring the quality of results. Only three discrepancies were observed and after careful re-checking, the results of the senior microscopist were taken as final. The slides were examined within 30–60 min of preparation for not missing the hookworms.

## Results

A total of 1021 individuals participated in the study and provided stool sample for parasitic examination, of which 518 (50.7%) were males and 503 (49.3%) were females. The age of the study participants ranged from 1 to 90 years, with the median age of 13 (Inter-quartile range: 6–27). Majority (40.7%) of the study participants were in the age group of 6–19 years. Results of the study showed that 532 individuals were infected with at least one parasite, providing the overall prevalence of 52.1% among the studied communities. As indicated in Table 1, generally 10 different parasite species were identified in the present study. The predominant STHs infection was hookworm, with a prevalence of 44.1%, followed by *A. lumbricoides* (10.3%), and *T. trichiura* (4.4%). The prevalence of the rest parasites are as follows: *H. nana* (2.1%), Taenia spp (1.4%), *S. stercolaris* (1.2%), *E. vermicularis* (1.1%), *E. histolytica/dispar* (0.7%), *S. mansoni* (0.6%) and *G. lamblia* (0.1%) (Table 1).

**Table 1:** Age-sex distribution of the intestinal parasitic infection and polyparasitism among the study participants

Variables	Positives	Frequency of infection with different species of parasites and polyparasitism										
		Nematodes					Cestodes		Protozoa		Trematodes	PP
		HW	AL	TT	SS	EV	TS	HN	EH	GL	SM	
Gender												
Male	266	227	46	28	7	7	7	10	4	1	5	66
Female	266	223	59	17	5	4	7	11	3	0	1	61
Total	532 (52.1%)	450 (44.1%)	105 (10.3%)	45 (4.4%)	12 (1.2%)	11 (1.1%)	14 (1.4%)	21 (2.1%)	7 (0.7%)	1 (0.1%)	6 (0.6%)	127 (12.4%)
*p*-value	0.624	0.869	0.134	0.115	0.596	0.823	0.956	0.773	-	-	-	0.766
Age groups (years)												
≤ 15	271	224	65	30	6	10	5	14	5	1	2	76
> 15	261	226	40	15	6	1	9	7	2	0	4	51
Total	532 (52.1%)	450 (44.1%)	105 (10.3%)	45 (4.4%)	12 (1.2%)	11 (1.1%)	14 (1.4%)	21 (2.1%)	7 (0.7%)	1 (0.1%)	6 (0.6)	127 (12.4%)
*p*-value	0.001	0.001	0.179	0.131	0.443	0.274	0.129	0.308	-	-	-	0.319

Key: *HW* Hookworm*, AL Ascaris lumbricoides, TT Trichuris trichiura, SS Strongyloides stercoralis, EV Enterobius vermicularis, TS* Taenia spp*, HN Hymenolepis nana, EH Entamoeba histolytica, GL Giardia lamblia, SM Schistosoma mansoni, PP* Polyparasitism

Regarding infection intensity of STH and *S. mansoni* by Kato-Katz, the results revealed that all recorded infections were categorized as “light” except two *A. lumbricoides* cases which were “heavy”. Table 1 shows that there was no significant difference in prevalence of intestinal parasites among male and female participants (*p* = 0.624). However, the infection rate significantly differed for the age groups of the study participants; individuals above 15 years of age were found to have higher prevalence of intestinal parasites (OR: 1.5, 95% CI (1.1, 1.9). Specifically, the distribution of hookworm among the participants was significantly higher among individuals above 15 years of age (*p* = 0.001). Further analysis, as represented in Table 2, revealed that infection with at least one parasitic species significantly differed among the age groups of female participants (*p* = 0.004) but there was no significant difference among the age groups of male participants (*p* = 0.089). Moreover, females in the reproductive age group had higher odds of infection with intestinal parasites than females under 15 years of age (OR: 1.7, 95% CI (1.2, 2.4)).

**Table 2:** Association of sex and age group with Intestinal parasites infection status of the study subjects

Sex and age group	No examined	Infected (%)	OR (95%CI)	*p*-value
Female				
≤15	270	127 (47.1)	1	0.004
>15	233	139 (59.6)	1.7 (1.2, 2.4)	
Total	503	266 (52.8)		
Male				
≤15	299	144 (48.2)	1	0.089
>15	219	122 (55.7)	1.4 (0.9, 1.9)	
Total	518	266 (51.4)		

The overall prevalence of polyparasitism observed among the study participants was 12.4% (127/1021). Among individuals positive for any parasites, 405 (76.1%), 114 (21.4%), and 13 (2.5%) were infected with one, two and three species of parasites, respectively. As indicated in Table 3, hookworm and *A. lumbricoides* were the most frequently recorded combination both as double infection (52 of 114) and triple infection with another parasite (frequency of 10 of 13). Furthermore, hookworm, *T. trichiura,* and *A. lumbricoides* were the predominant combination observed in triple infections (frequency of 5 of 13), followed by hookworm, *T. trichiura* and *H. nana; and* hookworm, *T. trichiura* and *S. stercolaris* (frequency of 2 of 13 for each).

**Table 3:** Age-sex distribution of the parasites combination (polyparasitism) among the study participants

Polyparasitism type	Frequency	Gender		Age group (years)	
		M	F	≤15	>15
Double infection					
HW + AL	52	24	28	32	20
HW + TT	11	7	4	7	4
AL + TT	8	5	3	5	3
HW + EV	6	3	3	3	3
HW + HN	6	4	2	4	2
HW + SS	6	4	2	4	2
HW + TS	8	4	4	2	6
HW + EH/D	4	1	3	1	3
AL + SM	3	2	1	2	1
HW + SM	1	1	-	-	1
TT + SM	2	2	-	1	1
TT + SS	2	1	1	2	-
TT + HN	1	-	1	1	-
TT + EV	1	1	-	1	-
AL + SS	1	-	1	-	1
AL + EH/D	1	-	1	1	-
AL + EV	1	-	1	-	1
Triple infection					
HW + AL + TT	5	2	3	4	1
HW + TT + HN	2	1	1	2	-
HW + AL + SS	2	1	1	2	-
HW + AL + TS	1	1	-	-	1
HW + AL + EV	1	1	-	1	-
HW + AL + HN	1	-	1	1	-
HW + TT + TS	1	1	-	1	-

Key: *HW* Hookworm*, AL A. lumbricoides, TT T. trichiura, EV E. vermicularis, TS* Taenia species*, HN H. nana, SS S. stercolaris, EH/D E. histolytica/dispar, SM S. mansoni*

Overall, 6 cases of intestinal schistosomiasis (*S. mansoni*) were reported in this study with a prevalence of 0.6%. All of the schistosomiasis cases were found as part of polyparasitic infection. Out of 518 male participants, 66 (12.7%) of them were found to have polyparasitism while 61(12.1%) of 503 female participants had polyparasitism. Similarly, out of 569 individuals ≤15 years, 76 (13.4%) of them were found to have polyparasitism, while 51(11.3%) of 452 individuals >15 years had polyparasitism. The distribution of polyparasitism among the study participants showed no significant difference with gender (*p* = 0.766) and across the age groups (*p* = 0.319) (Table 1).

## Discussion

The present study has attempted to assess the distribution of IPIs and polyparasitism among the study subjects. Although lower when compared to the previously reported prevalence of 83% from urban dwellers of Jimma zone [19], the current study reported still a high overall prevalence of IPIs (52.1%). This difference might be attributed to time gap, because the previous study was conducted in 2004. Moreover, Jimma zonal health department successive reports since 2005 indicated much improvement in sanitation and personal hygiene through community mobilization and the extensive effort of health extension workers. Yet, the observed high prevalence of IPIs and polyparasitism indicates that communities of Gilgel Gibe dam area resides in heavily contaminated environment with parasites. It is also lower than the prevalence of 62.3% reported from Northwest Ethiopia [20], 72.9% from Azezo [21], and 83.8% from Southeast of Lake Langano [22]. However, it is higher than what was reported from elsewhere [23–26]. These differences could be due to geographical and climatic variations, and as well might be due to socio-demographic and socio-economic differences of the study populations.

In this study, there was no significant difference in prevalence of any IPIs among the gender. This is similar to what was reported from elsewhere [25]. The predominant parasite infection found in this study was hookworm (44.1%). This could be due to the fact that our study participants are from rural community and during study period we observed that majority of the participants are working in the open farm for living; and this environment might favors the easy transmission of hookworm. This finding is consistent with the study conducted in rural Côte d’Ivoire, in which hookworm was the predominant STHs in the community with a prevalence of 45% [27]. In the present study, significant association was found between hookworm infection and age group. Older individuals were more infected with hookworm species than the younger population. It confirms the hypothesis that the probability of acquiring hookworm infection increases with age as the activity outside the household increases with age. This is consistent with the findings reported from elsewhere [25, 27].

Hookworm was predominantly found among individuals older than 15 years of age. This might have a negative implication for mass drug administration (MDA) which specifically focuses on school children (6–15 years of age) as majority of the infection is contributed by older individuals. Perhaps the MDA should include people older than 15 years of age for better control of the diseases. The low prevalence of *S. stercolaris* and *E. vermicularis* might be attributed to the employed technique used for screening and the heterogeneity of the study participants with respect to age. In the present study, specific techniques like scotch tape test and Harada Mori which are known to yield better results were not used as indicated in the limitation section.

The results of this study revealed that polyparasitism was prevalent among the study population with an overall prevalence of 12.4%. These results are based on low sensitive diagnostic methods (direct wet mount and Kato-katz thick smear) and single stool sample examination. In addition to the limitation of those diagnostic methods, considering other confounding factors like day-to-day variation in eggs or cysts output of some of the parasites, the ‘true prevalence’ of polyparasitism is likely higher than what is reported. Moreover, our finding is in agreement with the finding of similar previous works [12, 19, 25, 28–30] where polyparasitism is commonly reported, although the reported prevalence varies considerably from place to place being either higher or lower than what we have reported here. In general, this variation could be attributed to different factors such as ecological and geographical location, climate and nature of soil, the segments of the populations studied, the diagnostic methods employed and other related socio-demographic and socio-economic factors.

The distribution of polyparasitism was not significantly associated with gender. This was similar with the finding of a study published elsewhere [24]. The most frequently encountered parasites combination, in cases of polyparasitism, was the concurrent infection with hookworm and *A. lumbricoides,* which accounted for 48.8% (62/127) co-existing as (double and triple infection) among the study participants (Table 3). This is consistent with other studies conducted elsewhere [26, 31]. This might be due to the residential area of the communities studied where outdoor activity is common and similarity of the two parasites in contaminating the soil or environment that could serve as source of infection. Similarly, we observed significant association between *A. lumbricoides* and *T. trichiura* in the present study which is in agreement with other studies [27, 32, 33] making possible the contribution of oro-fecal route transmission of both parasites. In general as indicated in Table 3, our study has shown the co-infection of the different parasites in the studied communities particularly indicating the overlap of different STHs among themselves and with other IPIs indicating that the communities are at risk of acquiring multiple-parasites infections and calling for integrated control approaches.

Furthermore, this finding also reported 0.6% of *S. mansoni*. Given that there was no evidence of schistosomiasis case report around Gilgel Gibe dam community coupled with the newly formed body of water due to the hydroelectric dam, the authors believe that this finding highlights the potential risk of schistosomiasis as a threat to that particular community in the near future, even if the prevalence appears very low for now. Thus, regular survey for schistosomiasis as well as close follow-up for malocological evidence is indicated to prevent the possibility of potential raise of schistosomiasis cases.

## Conclusion

Our study highlighted the prevalence of intestinal polyparasitism among residents around Gilgel Gibe dam. Hookworm infection was the predominant STHs in the area. Age group was significantly associated with infection. The distribution of hookworm was significantly high among people older than 15 years of age. This might hamper the outcome of MDA, which focuses on school children, suggesting the need to include individuals other than school children. The most frequent combination of parasites in polyparasitized individuals was hookworm and *A. lumbricoides*. Further study is needed to investigate risk factors and predictors of polyparasitism with STHs in the study area. A study designed to investigate the effect of polyparasitism on individuals should be in place as well. Integrated control strategies which consider, community health education, personal hygiene and improved sanitation including MDA that targets the whole community instead of only certain segments of populations need to be considered for effective control of IPIs.

### Strength and limitation of the study

In contrast to many studies focusing on narrow age group, this study focused on the entire population having an age range of 1 to 90 years and used large sample size. However, due to logistic reasons, the study did not consider all possible available stool sample examination techniques to exhaustively determine all intestinal parasites with maximum sensitivity and was limited to use of only single stool sample. Therefore, the reported results in this study might be much less than the ‘true prevalence’ among the studied communities. Additionally, although we did reading of kato-katz smears within 30 min for maximum recovery of hookworms, we did not carry further examination to identify hookworm species.
